# Objective assessment of motor fatigue in multiple sclerosis using kinematic gait analysis: a pilot study

**DOI:** 10.1186/1743-0003-8-59

**Published:** 2011-10-26

**Authors:** Aida Sehle, Annegret Mündermann, Klaus Starrost, Simon Sailer, Inna Becher, Christian Dettmers, Manfred Vieten

**Affiliations:** 1 Division of Sport Science, Universität Konstanz, Konstanz, Germany; 2 School of Physiotherapy, University of Otago, Dunedin, New Zealand; 3 Kliniken Schmieder Allensbach, Allensbach, Germany; 4 Department of Politics and Public Administration, University of Konstanz, Konstanz, Germany; 5 Kliniken Schmieder Konstanz, Konstanz, Germany

## Abstract

**Background:**

Fatigue is a frequent and serious symptom in patients with Multiple Sclerosis (MS). However, to date there are only few methods for the objective assessment of fatigue. The aim of this study was to develop a method for the objective assessment of motor fatigue using kinematic gait analysis based on treadmill walking and an infrared-guided system.

**Patients and methods:**

Fourteen patients with clinically definite MS participated in this study. Fatigue was defined according to the Fatigue Scale for Motor and Cognition (FSMC). Patients underwent a physical exertion test involving walking at their pre-determined patient-specific preferred walking speed until they reached complete exhaustion. Gait was recorded using a video camera, a three line-scanning camera system with 11 infrared sensors. Step length, width and height, maximum circumduction with the right and left leg, maximum knee flexion angle of the right and left leg, and trunk sway were measured and compared using paired t-tests (α = 0.005). In addition, variability in these parameters during one-minute intervals was examined. The fatigue index was defined as the number of significant mean and SD changes from the beginning to the end of the exertion test relative to the total number of gait kinematic parameters.

**Results:**

Clearly, for some patients the mean gait parameters were more affected than the variability of their movements while other patients had smaller differences in mean gait parameters with greater increases in variability. Finally, for other patients gait changes with physical exertion manifested both in changes in mean gait parameters and in altered variability. The variability and fatigue indices correlated significantly with the motoric but not with the cognitive dimension of the FSMC score (R = -0.602 and R = -0.592, respectively; P < 0.026).

**Conclusions:**

Changes in gait patterns following a physical exertion test in patients with MS suffering from motor fatigue can be measured objectively. These changes in gait patterns can be described using the motor fatigue index and represent an objective measure to assess motor fatigue in MS patients. The results of this study have important implications for the assessments and treatment evaluations of fatigue in MS.

## Background

Multiple Sclerosis (MS) is a chronic autoimmune disease of the central nervous system characterized by inflammation, demyelization and destruction of axons and neurons, and by gliosis. MS is the most common neurological disorder in younger adults with a prevalence of 30-110 per 100, 000 adults [1,2]. In Germany alone, approximately 130, 000 patients suffer from multiple sclerosis [1]. Multiple sclerosis comprises a variety of symptoms including central paresis, spasticity, paraesthesia, ataxia, dysarthria, visual impairment, cognitive dysfunction and urinary and bowel dysfunction [3]. However, the most common and most debilitating symptom [4-6] experienced by 87-92% of all persons affected by MS is fatigue, recently termed 'pathological exhaustion' [7], which is defined as 'a subjective lack of physical or mental energy that is perceived by the individual or caregiver to interfere with activities of daily living' [8].

The pathophysiology of fatigue in MS is still poorly understood and the success rates of available treatments are low. Fatigue is typically exacerbated by exertion and by heat, where the latter is known as the Uhthoff phenomenon [9]. Use-dependent conduction block has been proposed as a likely mechanism of fatigue in MS [10]. It has been suggested that activity results in axonal hyperpolarization [11] and that conduction blocks may be induced by depletion of axonal energy supply or by inflammatory mediators [12,13]. Other changes associated with fatigue in MS patients are increased and extensive cortical activation (including that of non-motor cortical areas) and reduced cortical inhibition during simple motor tasks [14,15], and white and grey matter volume loss [16]. Current management of fatigue in MS includes physical-based options (such as aerobic exercise, energy conservation strategies, and psychological and dietary interventions) [17-19], cooling [20,21], measures to ameliorate conduction block [22] and the use of other pharmacological agents [23,24].

The evaluation of treatment efficacy and a patient's ability to better perform occupational tasks require a valid and reliable assessment of fatigue in MS where patients may suffer from cognitive or from motor fatigue of from both. Current clinical methods for the assessment of motor fatigue in MS are self-reported instruments for the assessment of subjective fatigue or the perception that more effort is required to perform a task. These instruments include the Fatigue Severity Scale (FSS) [25], the Fatigue Impact Scale (FIS) [26], the Fatigue Descriptive Scale (FDS) [27], and a Visual Analogue Scale (VAS) [28]. While most of these instruments have adequate validity and reliability [26,28,29], they all rely on subjective reporting and are unable to differentiate between inability and reluctance to generate or maintain the required force. While recent technological developments [30] are promising for measuring fatigue objectively, they do not provide information on patient function.

Clinically, motor fatigue can be defined as a reduction in maximal walking distance that cannot be explained by the degree of paresis, ataxia or spasticity. Many patients with motor fatigue demonstrate a gait pattern that is initially close to normal, although angular exertions may be statistically smaller [31], but distinctly different from normal when they are exhausted. Patients are generally able to clearly describe the changes in their gait pattern, such as, for instance, one of their feet starting to drop, one leg being dragged or becoming unsteady. Hence, recording patients' perception of their function or change in function provides critical information for assessing a patient's status. Interestingly, the maximum walking distance to exhaustion on a treadmill at standardized conditions without prior exertion and after a full night's rest appears to be constant for each individual [32] suggesting a physical cause for their perceived exhaustion. Consequently, it is possible that abnormalities will only manifest in a neurological exam following physical exhaustion. Hence, objective assessment of these functional alterations during an exertion test may provide insight into underlying neurological changes associated with MS and form the foundation for determining limitations of a patient's working capacity that may warrant additional or alternative treatment or early retirement.

The purpose of this study was to develop an objective tool for the assessment of motor fatigue in MS, the *fatigue index*. It was hypothesized that specific gait parameters including step length, width and height, bilateral circumduction, bilateral knee flexion angle and medio-lateral sway change during the exertion test, and that the variability of the step cycle is different after compared to prior to the exertion test.

## Methods

From March to April 2009, fourteen patients with definite MS were screened in a neurological rehabilitation clinic for complaints about motor fatigue and having a limited maximal walking distance. The study was approved by the Institutional Review Board and was conducted in accordance with the Declaration of Helsinki. The duration of one data collection session was one hour.

### Subjects

Fourteen patients participated in this study after giving informed consent (nine females and five males; age: 42 ± 7.6 years; height: 1.71 ± 0.09 m; mass: 76.1 ± 19.2 kg). Patients' impairment ranged from minimal to moderate signs of impairment (Expanded Disability Status Scale (EDSS): 3.6 ± 1.33; range: 1.0-5.5). Time since onset of symptoms was 7.5 ± 5.7 years and time since diagnosis 5.0 ± 4.4 years. Maximal walking distance until exhaustion was 362 ± 439 m (63-1524 m).

### Fatigue questionnaire

Fatigue was rated using the self-administered Fatigue Scale for Motor and Cognition (FSMC). The scale was recently developed and evaluated [33] and found to be sufficiently sensitive to discriminate between motor and cognitive fatigue. Ten questions relate to motor fatigue and ten to cognitive fatigue. Scores between 22 and 26 points indicate light motor fatigue, scores between 27 and 31 points indicate moderate fatigue, and scores of 32 points or higher indicate severe fatigue. Corresponding ranges for cognitive fatigue are 22-27, 28-33 and ≥34 points.

### Physical Exertion test

Each patient participated in a physical exertion test on a treadmill. For this test, patients walked on a treadmill until they experienced complete exhaustion. Patients were wearing a safety harness to prevent falling. The speed of the treadmill was set to a subject-specific comfortable walking speed and kept constant throughout the test. During the test, patients were repeatedly asked to rate their physical exhaustion on a scale from 1 (not exhausted at all) to 10 (unable to continue the test). The physical exertion test was stopped one minute after the patient seriously requested to stop or to rest (completely exhausted; mean exhaustion score: 6.1 ± 2.4).

### Gait recording

**Figure 1:**
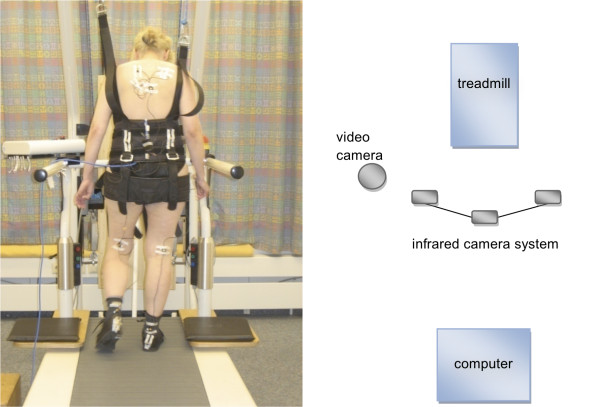
**Test set-up**. Patients wore safety harness during all tests to prevent injury by potential falls. The infrared camera system and the video camera were positioned posterior of the patient behind the treadmill. The acquisition computer was operated by one tester and placed behind the cameras to allow for visual observation of all tests.

Gait data was recorded using the wireless AS200 system (80 Hz; LUKOtronic, Lutz Mechatronic Technology e.U., Innsbruck, Austria) consisting of a three line-scanning camera system and 11 active infrared markers with a 2-mm accuracy. The markers are connected by cable to a unit worn on a belt. The camera unit was positioned posterior of the patient behind the treadmill (Figure 1). The system was synchronized with a standard video camera (Digital Ixus 65, Canon Inc., Tokyo, Japan). Eleven active infrared markers were attached to the patient's body: bilaterally on the shoes on top of the calcaneus; bilaterally on the Achilles tendon at the level of the ankle; bilaterally on the posterior aspect of the knee; bilaterally on the belt at the highest point of the ilium; on the spine at the level of the sternum; bilaterally centered on Margo medialis.

After a patient reached comfortable walking speed, three dimensional marker data and video images were recorded for one minute at the beginning of the test (t_1_) and for one minute when patients stated that they could no longer walk and were completely exhausted (t_2_). Following this statement, the patient had to walk for one more minute, and data for this minute was recorded (t_2_). The current physical exhaustion at each of the recordings was charted on the physical exhaustion scale (see above) before and after physical exertion. Processing time of gait data was one hour per subject.

### Pathological diagnostic criteria (gait abnormalities)

Step length, step width, step height, maximum circumduction with the right and left leg, maximum knee flexion angle of the right and left leg, and medio-lateral sway of the upper body were calculated for each step using the three-dimensional coordinates of the infrared markers. Mean and standard deviations for each parameter and time interval were calculated for each patient and used for further analysis. Significant changes in the mean and standard deviations of these parameters were used as probable indicators of fatigue. It was assumed that a patient's gait pattern at the rested state corresponds to their "normal" gait pattern. Therefore, the changes in gait parameters after physical exertion can be regarded as pathological, although the direction of changes was irrelevant. The fatigue index comprised components of mean gait changes and changes in variability and was defined as

$$\begin{array}{c} index_{fatigue}=\frac{1}{2}\cdot \left(index_{mean}+index_{\text{var}iability}\right)\\ =\frac{1}{2}\cdot \left(\frac{N_{\text{si}gnificant\text{\_}mean\text{\_}changes}}{N_{gait\text{\_}parameters}}+\frac{N_{\text{si}gificant\text{\_}SD\text{\_}changes}}{N_{gait\text{\_}parameters}}\right) \end{array}$$

where *N_significant_mean_changes _*was the number of parameters that had a significant mean change from t_1 _to t_2_, *N_significant_SD_changes _*was the number of parameters that had a significant SD change from t_1 _to t_2 _and *N_gait_parameters _*was the number of gait parameters. Step length, step width, step height are global (non-side-specific) measures, and differences in these parameters can originate from differences in the left leg, right leg or both legs. Hence, these global gait parameters were weighted with a factor 2 and the side-specific parameters right and left circumduction and right and left knee flexion angle were weighted with a factor 1. Possible values for the fatigue, mean index and variability indices are between 0 and 1, respectively.

### Statistical Analysis

All statistical tests were performed using StatFree Version 4.4.2.2 (VietenDynamics) and Stata Version 10.1 (StatCorp LP, College Station, Texas, USA). Descriptive analyses of numerical parameters included mean, median, minimum and maximum, and distribution and standard deviation. All parameters were tested for normal distribution. Differences in normally distributed parameters between t_1 _and t_2 _were detected using Student's t-tests for paired samples. Differences in non-normally distributed parameters between t_1 _and t_2 _were detected using Wilcoxon signed-rank tests. Differences in parameter variability between t_1 _and t_2 _were detected using the standard deviation test (SD test). Bonferroni adjustment was applied to account for multiple comparisons, and the significance level for all statistical tests was set a priori to α = 0.005. Bivariate Pearson correlation coefficients were used to detect significant associations between the components of the fatigue index, the dimensions of FSMC and the distance walked during the physical exertion test (α = 0.05).

## Results

**Figure 2:**
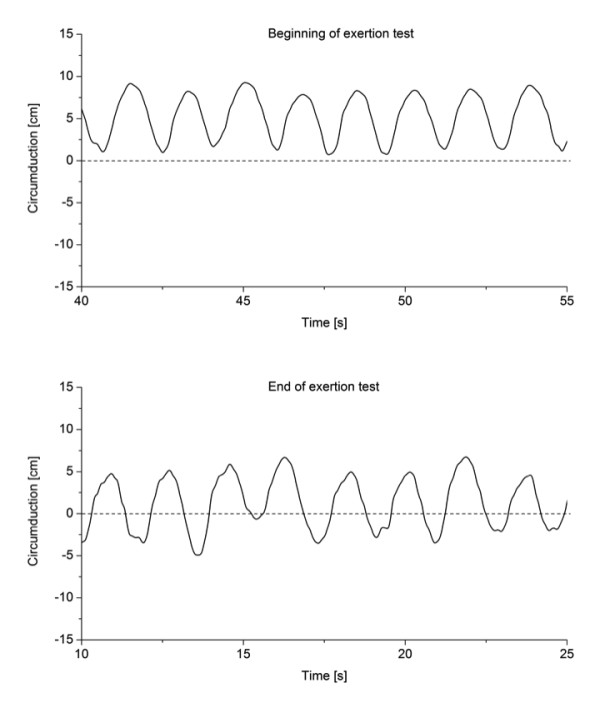
**Circumduction of the right leg in a 15-sec interval during the first (top graph) and last (bottom graph) minute of the physical exertion test for patient 9**.

**Figure 3:**
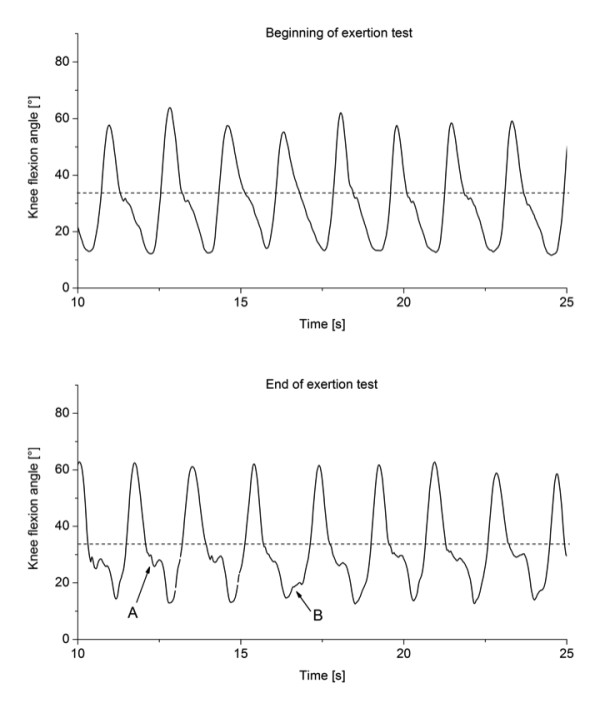
**Knee flexion angle in a 15-sec interval during the first (top graph) and last (bottom graph) minute of the physical exertion test for patient 5**. A--additional variability during knee extension; B--additional variability close to full knee extension.

The fatigue index for this patient group ranged from 0.33-0.92, the mean index ranged from 0.00-0.92 and the variability index ranged from 0.25-0.92 (Table 1). Clearly, for some patients the mean gait parameters were more affected than the variability of their movements while other patients had smaller differences in mean gait parameters with greater changes in variability. Finally, for other patients gait changes with physical exertion manifested in both changes in mean gait parameters and in altered variability. For instance, one patient (patient 9) showed relatively regular patterns of circumduction with their right leg at the beginning of the physical exertion test with a shift in circumduction to smaller values and more variable wave patterns at the end of the physical exertion test (Figure 2). Another patient (patient 5) showed similar mean values for their knee flexion angles during one minute but had clear irregularities in their pattern manifesting as more irregular knee extension movements and additional irregularities close to full knee extension (Figure 3).

**Table 1:** Fatigue index with sub-indices mean and variability for all patients

*Patient ID*	*Index_mean_*	*Index_variability_*	*Index_fatigue_*
1	0.00	0.67	0.33
2	0.83	0.67	0.75
3	0.75	0.58	0.67
4	0.42	0.42	0.42
5	0.58	0.58	0.58
6	0.42	0.25	0.33
7	0.67	0.42	0.54
8	0.58	0.67	0.63
9	0.58	0.50	0.54
10	0.67	0.50	0.58
11	0.75	0.33	0.54
12	0.92	0.92	0.92
13	0.58	0.33	0.46
14	0.50	0.58	0.54

Mean	0.59	0.53	0.56
SD	0.22	0.17	0.16

**Figure 4:**
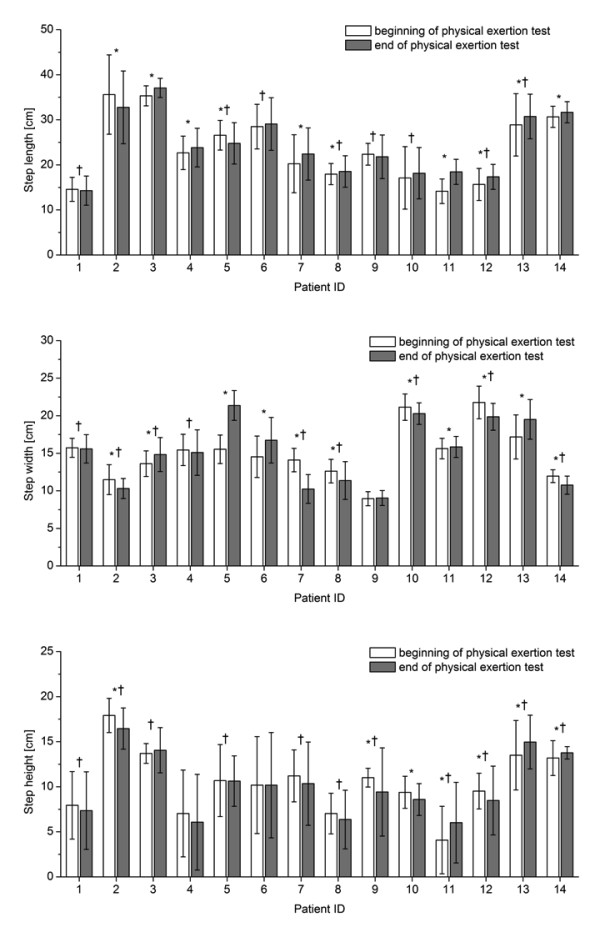
**Mean (1SD) step length, width and height for each patient during one minute of treadmill walking at the beginning and at the end of the physical exertion test, respectively**. * indicates significant differences between mean values at the beginning and end of the test; † indicates significant differences between the standard deviations at the beginning and end of the test (P < 0.005).

**Figure 5:**
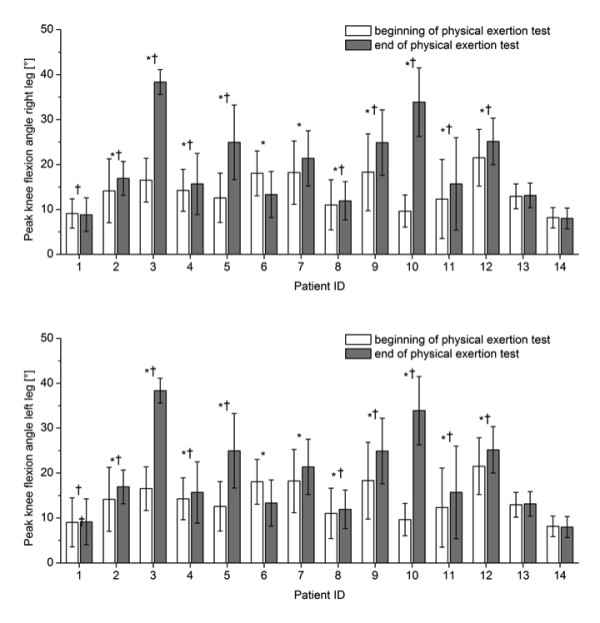
**Mean (1SD) peak knee flexion angle for the right and left leg for each patient during one minute of treadmill walking at the beginning and at the end of the physical exertion test, respectively**. * indicates significant differences between mean values at the beginning and end of the test; † indicates significant differences between the standard deviations at the beginning and end of the test (P < 0. 005).

**Figure 6:**
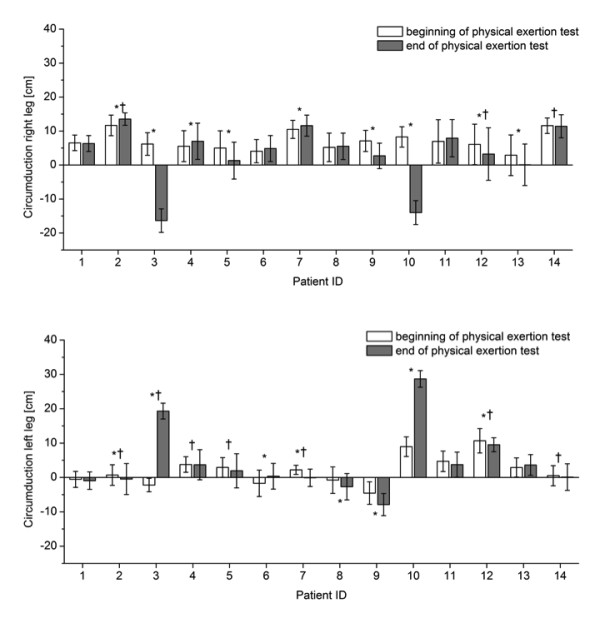
**Mean (1SD) circumduction for the right and left leg for each patient during one minute of treadmill walking at the beginning and at the end of the physical exertion test, respectively**. * indicates significant differences between mean values at the beginning and end of the test; † indicates significant differences between the standard deviations at the beginning and end of the test (P < 0. 005).

**Figure 7:**
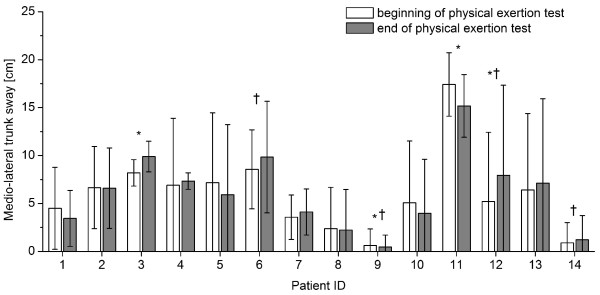
**Mean (1SD) medio-lateral trunk sway for each patient during one minute of treadmill walking at the beginning and at the end of the physical exertion test, respectively**. * indicates significant differences between mean values at the beginning and end of the test; † indicates significant differences between the standard deviations at the beginning and end of the test (P < 0. 005).

The gait parameters that showed significant differences with fatigue for most patients were step length, width and height (Figure 4) followed by knee flexion angle (Figure 5) and circumduction (Figure 6). The gait parameter that showed significant differences with fatigue for the least number of subjects was trunk sway (Figure 7).

The variability index and the fatigue index correlated significantly with the overall FSMC and with the motoric dimension of the FSMC, respectively (Table 2). In contrast, the mean index did not correlate significantly with any of the FSMC dimensions. While the fatigue index correlated with both the mean index and the variability index, the mean index and the variability index did not correlate significantly. None of the components of the fatigue index correlated with the distance walked during the physical exertion test. All dimensions of the FSMC correlated significantly with each other. The mean overall, cognitive and motoric FSMC scores were 64.3 ± 19.3, 26.6 ± 12.3 and 37.7 ± 8.3 points, respectively (indicating severe global fatigue, light cognitive fatigue and severe motor fatigue, respectively).

**Table 2:** Cross-correlations (Pearson's correlation coefficient, P-value) between dimensions of the fatigue index, dimensions of the Fatigue Scale for Motor and Cognition (FSMC) and distance walked during the physical exertion test

*R* *P-value*	*index_mean_*	*index_variability_*	*index_fatigue_*	*FSMC_overall_*	*FSMC_cognitive_*	*FSMC_motoric_*	*distance walked*
*index_mean_*	1						
*index_variability_*	0.209 0.473	1					
*index_fatigue_*	**0.835** **< 0.001**	**0.713** **0.004**	1				
*FSMC_overall_*	-0.209 0.473	**-0.560** **0.037**	-0.465 0.094	1			
*FSMC_cognitive_*	-0.092 0.753	-0.473 0.087	-0.331 0.248	**0.958** **< 0.001**	1		
*FSMC_motoric_*	-0.350 0.220	**-0.602** **0.023**	**-0.592** **0.026**	**0.906** **< 0.001**	**0.747** **0.002**	1	
*distance walked*	0.366 0.198	0.277 0.338	0.421 0.134	**-0.535** **0.049**	-0.461 0.097	**-0.562** **0.037**	1

Significant correlations (P < 0.05) are shown in bold font.

Overall, seven of the eight gait parameters changed significantly between t_1 _and t_2 _for this group of patients (p < 0.001; Table 3). When fatigued, patients walked on average with longer step lengths, smaller circumduction with their right leg, greater circumduction with their left leg, flexed their knees more and swayed their upper bodies more than prior to exertion. The SD-tests revealed that the variability of steps between t_1 _and t_2 _increased for seven gait parameters with increasing exhaustion of the patients (p < 0.003; Table 1). Following exertion, the variability of the significant gait parameters increased by 9-121% compared to prior to exertion. On average, the mean index and the variability index showed comparable values (Table 1).

**Table 3:** Results of the t-Test and SD-Test comparing eight gait parameters between t_1 _and t_2 _(N = 14)

*Gait parameters*	*Mean (t_1_)*	*Mean (t_2_)*	*Significance* *t-Test*	*Std. Dev. (t_1_)*	*Std. Dev. (t_2_)*	*Significance* *SD-Test*
Step width [cm]	15.3	15.5	0.032	3.9	4.4	< 0.001
Step height [cm]	10.1	9.8	n.s.	4.7	5.1	0.002
Step length [cm]	23.6	24.3	0.005	7.4	6.9	n.s.
Circumduction right leg [cm]	6.8	1.6	< 0.001	4.7	10.4	< 0.001
Circumduction left leg [cm]	1.6	5.5	< 0.001	4.9	10.4	< 0.001
Knee flexion angle right leg [°]	12.8	20.5	< 0.001	6.4	11.7	< 0.001
Knee flexion angle left leg [°]	12.7	17.8	< 0.001	7.9	10.8	< 0.001
Sway [cm]	3.4	3.9	< 0.001	5.4	6.0	< 0.001

n.s.--not significant at α = 0.05

## Discussion

According to guidelines proposed by the MS Council for Clinical Practice Guidelines in 1998, fatigue is defined as „a *subjective *lack of physical and/or mental energy that is perceived by the individual or caregivers to interfere with usual and desired activities" [34]. Within this definition, the term *subjective *implies that fatigue is not measurable, may be psychogenic or not even exist. However, the results of this study clearly showed--despite pre-determined constant walking speed--(a) that fatigue in MS patients manifests as changes in gait patterns and (b) that some changes in gait patterns associated with fatigue are consistent across a group of patients suffering from MS. Hence, the results of this study provide evidence for the existence of motor fatigue and suggest that motor fatigue is a pathophysiological phenomenon.

The significant correlations of the fatigue index with its subcategories mean index and variability index and the lack of statistical significant correlations between these two subcategories suggest that both the mean and variability index described two different phenomena. Hence, both subcategories are important measures for motor fatigue in MS. In addition, the significant correlation of the variability and fatigue indices with the motoric dimension of the FSMC but not with its cognitive dimension supports the specificity of the fatigue index for the motoric aspect of fatigue in multiple sclerosis.

Interestingly, the fatigue index correlated negatively with the FSMC. The FSMC is a self-administered questionnaire, and data obtained with the FSMC may be distorted by overestimation because of a deficient self-awareness or underestimation because of depression. Depression is a well-known confounding factor of the FSMC [33]. This discrepancy highlights the urgent need for an objective marker of fatigue. In addition, while the FSMC measures the overall subjective status of a patient, the fatigue index describes the extent to which a patient's gait changes with fatigue. The results of this study suggest that gait patterns of patients with a poor overall subjective status will be affected less by fatigue than those of patients with a better overall subjective status. It is possible that gait patterns in patients with a poor overall subjective status are already compromised at the beginning of the fatigue test. This result suggests that comparing general gait patterns in MS patients to those of age-matched healthy subjects may provide additional objective information about a patient's functional status.

Individual results showed changes in variability of movement patterns with fatigue. Greater variability during knee extension and close to full extension in one patient (Figure 2) suggests disrupted motor coordination, which may be caused by additional activity of the antagonists or by insufficient force production by the agonists. For instance, patients with MS use excessive forces for daily tasks such as lifting and placing an object [35]. Thus, it is feasible that using excessive muscle force during daily activities such as walking may result in additional fatigue that manifests as increased variability of movement patterns.

Multiple reasons may be responsible for the changes in gait patterns observed with fatigue in MS patients. Patients in this study presented with slightly increased step length at the end of the physical exertion test, which--from a clinical perspective--is not typical for motor fatigue in MS patients. However, this change could be explained by the presence of muscle fatigue. Granacher et al. [36] previously showed that muscle fatigue generated by isokinetic contraction resulted in greater stride length in older healthy subjects while resulting in reduced stride length in younger subjects. Hence, it is possible that patients with MS suffer from an earlier on-set and faster rate of muscle fatigue compared to healthy control subjects. In addition, MS patients with greater fatigue have reduced isometric strength in the quadriceps muscle [37], which may represent compromised capacity to produce sufficiently large muscle moments about the joints of the lower extremities during walking.

Interestingly, functional imaging studies have reported increasing evidence that patients with MS experience greater cerebral activity during performance of motor and cognitive tests compared to normal volunteers [38,39]. Similar observations have been made in patients after manifestation of their first clinical symptom (clinically isolated syndrome, CIS) [40,41] and in patients without neurological deficits at the time of the functional imaging [42]. In addition, patients with a benign course of MS have shown increased cerebral activity [43] which may represent some form of compensation. In the late phase of MS (and with increasing fatigue) this mechanism of compensation is exhausted and compensatory cerebral activity is decreased [44,45]. However, while only few investigations have investigated a direct relationship between fatigue and functional imaging [15], stimulation studies have found that impaired central motor activation is involved in MS-fatigue [37]. Other studies [46] reported an increased central activation during fatiguing exercises probably reflecting an additional compensatory central activation. Thus, observed deterioration of gait parameters in exhausted patients could also reflect a breakdown of these compensatory mechanisms. In addition, the fact that patients with a progressive disorder such as multiple sclerosis show only small improvements in motor-evoked potential and maximum voluntary contraction using functional electrical stimulation [47] suggests compromised plasticity of their motor cortex and that their impaired motor activation is presumably associated with diminished muscle coordination. Hence, the gait changes observed following the physical exertion test in MS patients may stem from the combination of reduced muscle strength and diminishing coordination reflected in greater variability in movement patterns.

Individual gait changes with fatigue in MS patients are expected to be asymmetric, that is affecting either the left or the right side more, because typically disseminated regions are involved. Indeed, gait compensation with fatigue in this study population was asymmetric. However, the sidedness of these effects, that is circumduction with their right leg decreased substantially while circumduction with their left leg increased considerably, presumably occurred by chance. It can be assumed that in a larger study, differences in gait patterns with fatigue in MS patients would be asymmetric but not side-specific. In addition, it is possible that different symptomatology, such as spastic syndromes or ataxic disturbances, may be reflected in different changes in gait patterns.

Gait patterns of MS patients differ from those of healthy persons [31]. Kelleher et al. [31] reported reduced gait speed, reduced maximum hip and knee extension, ankle plantarflexion angle and propulsive force for MS patients compared to healthy persons and that these changes are more pronounced in more severely affected patients. Hence, the results of Kelleher et al. and those of this study suggest that fatigue in MS patients appears to amplify changes in gait patterns already present because of the disease. While the study sample in this study was rather small, it is possible that in the general MS population the extent of gait changes with fatigue is associated with the severity of symptoms. For instance, patients with greater perceived walking limitations have less movement counts from an accelerometer compared to patients with smaller walking limitations [48]. In addition, the results of this study showed that gait patterns generally become more variable or clumsier with fatigue. Such changes in gait patterns may generate other problems such as perception of instability or increased risk of falling. Thus, the changes in gait patterns observed in fatigued MS patients likely affect a patient's completion of daily activities.

Therefore, assessing changes in gait patterns using a physical exertion test and the fatigue index may be useful for the objective assessment of functional limitations associated with fatigue in MS patients and for evaluating rehabilitation programs aimed at improving patient function and reducing fatigue. However, the maximum distance walked during the exertion test should also be considered in the evaluation of such interventions. In addition, such an objective tool may be useful for differentiating between MS related motor fatigue and conditions that are unrelated to MS but may cause lack of energy (Table 4). Interestingly, only few subjects showed differences in trunk sway with fatigue, and hence the inclusion of this parameter in the fatigue index should be reconsidered. However, it is possible that trunk sway was restricted by the use of the safety harness in this group of patients. The influence of these factors should be examined in future studies. While obtaining gait data is more time-consuming than conventional assessment tools (i.e. questionnaires [26,27,29,33]) and requires specialized technical equipment, the information gained in this study is objective--and hence not affected by a patient's contorted self-awareness--and reliable. The latter is the prerequisite for obtaining meaningful data on a patient's physical status and may be particularly valuable for assessing a patient's ability to perform occupational tasks and consequently for determining a patient's entitlement for early retirement because of their disease. Comparing gait patterns in MS patients with and without fatigue and in healthy volunteers would allow for elucidation of the different dimensions, particularities and special features in gait patterns of fatigue in MS patients.

**Table 4:** Differential diagnosis of fatigue or causes of secondary weakness/tiredness in MS

*MS related causes for lack of energy*	*Non-MS related causes for lack of energy*
Depression	Depression
Nocturia	Thyroid function
Sleep disturbance	Anemia
Spasticity, paresis, uneconomic movement	Infection (bladder)
Lack of condition	Electrolytes
Side effects of medication (Liuresal, benzodiazepine etc.)	

## Conclusions

Distinct changes in gait patterns of MS patients were recorded through two identical tests before and following physical exertion. These changes in gait patterns can be expressed by the motor fatigue index and represent an objective measure to assess motor fatigue in MS patients. Assessing gait changes during a physical exertion test appears to be a useful experimental method for investigating different dimensions and pathomechanisms of fatigue in MS. In addition, an objective tool for assessing motor fatigue in MS is useful for a more precise diagnosis of motor fatigue in MS, for the design and evaluation of treatment and rehabilitation programs aimed at improving symptoms and for evaluating a patient's ability to perform occupational tasks.

## Competing interests

The authors declare that they have no competing interests.

## Authors' contributions

AS designed the study, collected, processed, analyzed and interpreted the data and outlined the manuscript. AM participated in data analysis, interpretation and presentation, and prepared the manuscript. KS and SS contributed to identifying pathological gait parameters and evaluated patient's videos. IB contributed to data processing and analysis. CD participated in study design, data interpretation and prepared the manuscript. MV conceived of the study, and participated in its design and coordination and helped draft the manuscript. All authors read and approved the final manuscript.
